# Supplementary material for: Efferocytosis perpetuates substance accumulation inside macrophage populations
Source: Proc Biol Sci. 2019 Jun 5;286(1904):20190730. doi: 10.1098/rspb.2019.0730 (PMC6571464; doi:10.1098/rspb.2019.0730)
Supplement: Methodology, Acknowledgments, Quantification of beads and lipids inside macrophages and Derivation and analysis of a coagulation-fragmentation model of substance accumulation in macrophages [file rspb20190730supp1.pdf]

# Methods, acknowledgments and supplementary material

This document contains the methods, data accessibility statement, acknowledgments and the supplementary material associated with the following article.

**Title:** Efferocytosis perpetuates substance accumulation inside macrophage populations

**Authors:** Hugh Z. Ford, Lynda Zeboudj, Gareth S. D. Purvis, Annemieke ten Bokum, Alexander E. Zarebski, Joshua A. Bull, Helen M. Byrne, Mary R. Myerscough and David R. Greaves.

**Journal:** Proceedings of the Royal Society B

**DOI:** 10.1098/rspb.2019.0730

# 1 Methods

## 1.1 Mathematical model

### 1.1.1 Model statement

The following system of non-linear differential equations (a coagulation-fragmentation model) was used to model the dynamic redistribution of beads inside macrophage populations via apoptosis, efferocytosis and division:

$$\frac{d}{dt}\phi_n = \underbrace{\eta \sum_{n'=0}^{n} \phi^{\dagger}_{n'}\, \phi_{n-n'}}_{\text{efferocytosis source}} \underbrace{-\eta\phi_n \sum_{n'=0}^{\infty} \phi^{\dagger}_{n'}}_{\text{efferocytosis sink}} \underbrace{-\beta\phi_n}_{\text{apoptosis sink}} \underbrace{+2\alpha \sum_{n'=n}^{\infty} \binom{n'}{n} \frac{\phi_{n'}}{2^{n'}}}_{\text{division source}} \underbrace{-\alpha\phi_n}_{\text{division sink}} \tag{1}$$

$$\frac{d}{dt}\phi^{\dagger}_n = \underbrace{-\eta\phi^{\dagger}_n \sum_{n'=0}^{\infty} \phi_{n'}}_{\text{efferocytosis sink}} \underbrace{+\beta\phi_n}_{\text{apoptosis source}} , \tag{2}$$

where $\phi_n = \phi_n(t)$ and $\phi^{\dagger}_n = \phi^{\dagger}_n(t)$ represent the number density of live and apoptotic macrophages that contain $n \geq 0$ beads at time $t \geq 0$. Equations (18) and (19) are closed by specifying initial distributions $\phi_n(0) = \psi_n$ and $\phi^{\dagger}_n(0) = \psi^{\dagger}_n$, $n \geq 0$. The apoptosis, efferocytosis and division rates were assumed to be independent of cellular bead content and assumed to occur with rates $\beta$ (per unit time), $\eta$ (per cell per unit time) and $\alpha$ (per unit time) respectively. We further assumed that bead numbers are conserved during apoptosis, efferocytosis and division. In more detail, we assume: (i) apoptosis produces one apoptotic cell with $n$ beads from one live cell with $n$ beads, (ii) efferocytosis transfers $n$ beads contained inside one apoptotic cell (consumed) to one live cell (consumer) that contains $n' \geq 0$ beads to produce one live cell that contains $n+n'$ beads and (iii) division produces two live macrophages (daughter cells) which contain $0 \leq n' \leq n$ and $n - n'$ beads from one live macrophage (parent cell) with $n$ beads. The mass action kinetics are shown in Figure 2A. A convolution source term models efferocytosis when all possible bead numbers inside live and apoptotic cells are accounted for. A binomial source term models cell division when each bead inside a parent cell is equally likely to transfer to either daughter cell upon division. The total number of live and apoptotic cells are defined as $N(t) = \sum_{n=0}^{\infty} \phi_n(t)$ and $N^{\dagger}(t) = \sum_{n=0}^{\infty} \phi^{\dagger}_n(t)$ respectively.

Several mathematical models were considered to test different mechanisms that might explain the experimental results. For example, the model produced under the assumption that dead cell constituents are shared among several macrophages (characteristic of late stage apoptosis and postapoptotic necrosis) fails to produce the large extent of substance accumulation and the large cell-to-cell variation which we see experimentally. Equations (18) and (19) was the simplest model which was consistent with our experimental observations.

### 1.1.2 Model solutions

We define the proportion of live and dead cells with $n$ beads by $p_n(t) \equiv \phi_n(t)/N(t)$ and $p^{\dagger}_n(t) \equiv \phi^{\dagger}_n(t)/N^{\dagger}(t)$ respectively. We make the simplifying assumption that efferocytosis is instantaneous ($\eta \to \infty$) such that

$p^\dagger_n(t) = p_n(t)$. With this rescaling and assumption, equations (18) and (19) reduces to:

$$\frac{d}{dt}p_n = \beta \sum_{n'=0}^{n} p_{n'}p_{n-n'} + 2\alpha \sum_{n'=n}^{\infty} \binom{n'}{n} \frac{p_{n'}}{2^{n'}} - (2\alpha+\beta)p_n \ . \tag{3}$$

The solution for the population size is $N(t) = N_0 e^{(\alpha-\beta)t}$ where $N_0$ is the initial number of cells.

Solutions to equation (36) are shown in Figure 2 for the case without cell division $\alpha = 0$ (Figure 2B) and with cell division $\alpha = \beta$ (Figure 2C). Here, we use an initial condition where every cell initially ($N_0$ cells) contains $n = 1$ bead ($p_n(0) = 1$ for $n = 1$ and $p_n(0) = 0$ for $n \neq 1$). With $\alpha = 0$, the population exponentially decays in size $N(t) = N_0 e^{-\beta t}$ and the proportion of cells with $n$ beads $p_n$ has the following geometric distribution (see Supplementary Material for details):

$$p_n = e^{-\beta t}\left(1 - e^{-\beta t}\right)^{n-1} \ . \tag{4}$$

With $\alpha = \beta$ the population size remains constant over time $N(t) = N_0$ and the proportion of cells with $n$ beads $p_n$ tends to an equilibrium state that satisfies the following relation:

$$3p_n = \sum_{n'=0}^{n} p_{n'}p_{n-n'} + 2\sum_{n'=n}^{\infty} \binom{n'}{n} \frac{p_{n'}}{2^{n'}} \ . \tag{5}$$

The forward Euler method [1] was used to numerically solve equation (36) with $\alpha = \beta$.

To compare the model to the experimental data shown in Figure 3, we assumed that bead-loaded macrophages stimulated with LPS and IFN$\gamma$ do not divide $\alpha = 0$ and die with rate $\beta = 1/60$ per hour (see Supplementary Material 2). The model prediction for the proportion of cells with $n$ beads ($p_n$) at $t = 24$ and 48 hours was found by numerically solving (forward Euler method [1]) equation (36) with an initial condition $p_n(0)$ equal to the experimental initial condition.

To compare the model to the experimental data shown in Figure 5, we assumed that macrophages stimulated with LPS and IFN$\gamma$ do not divide $\alpha = 0$ and die with rate $\beta = 1/80$ per hour (see Supplementary Material). We also assumed that macrophages stimulated with LPS and STPN do not divide $\alpha = 0$ and die with rate $\beta = 1/40$ per hour (see Supplementary Material). We generalise the number of beads per cell $n$ in our model to also represent the lipid content in units of the endogenous neutral lipid content (i.e. in cell membranes) per cell. We assume that every cell initially contains the same single unit of endogenous lipid such that $p_n(0) = 1$ if $n = 1$ and $p_n(0) = 0$ if $n \neq 1$. Thus the model prediction for the proportion of cells with lipid content $n$ ($p_n$) at $t = 24$ and 48 hours was found from equation (4).

## 1.2 Experimental methods

### 1.2.1 Cell culture

Hematopoietic stem cells were harvested from the femurbone marrow of mice. Animal studies were performed with local ethical approval from the Dunn School of Pathology Animal Welfare Ethical Review Board and according to the United Kingdom Home Office regulations (Guidance on the Operation of Animals, Scientific Procedures Act, 1986). C57BL/6 mice were obtained from the Biomedical Sciences Unit (Oxford, United Kingdom) and were housed in a 12-h light/dark cycle with free access to food and

water. These bone marrow cells were differentiated into macrophages by culturing for 7 days at 37°C and 5% $CO_2$ in 8mL high glucose Dulbecco's modified eagle media (DMEM) supplemented with 10% head-inactivated fetal bovine serum (FBS), 1% penicillin/streptomycin (P/S) and 10% supernatent derived from L929 fibroblasts (L929-condition media) as a source of macrophage colony-stimulating factor [2] in 100mm non-tissue culture treated Petri dishes (Thermo Fisher Scientific, Sterilin, UK). On day 5, 3mL of medium was removed and an additional 5ml of medium was added. Gentle scrapping was used to lift cells off dish surface. Cells were then counted and resuspended in DMEM at the desired cell concentration.

### 1.2.2 Bead accumulation

Cells were plated at $1 \times 10^5$ cells per well of a 4-well Nun-Tek™ Chamber slide™ (1.8 cm$^2$) or of a 24-well plate (2 cm$^2$) (Sigma-Aldrich, Gilligham, UK) with glass coverslip in high glucose DMEM supplemented with 10% FBS and 1% P/S and left at room temperature for 1 hour to adhere evenly across the plate. The cells were then incubated (37°C and 5% $CO_2$) for 6 hours and then exposed to $0.5 \times 10^5$ red and $0.5 \times 10^5$ blue 3 $\mu$m diameter latex polystyrene beads (Sigma-Aldrich, Gilligham, UK) such that there were 1 bead per cell. The beads were opsinised for 1 hour in 10%v/v human serum (Sigma-Aldrich, Gilligham, UK) obtained from human volunteers who had given informed consent and with ethical approval from the appropriate local ethics committee. Plates were centrifuged (400g for 1 min) so the beads were evenly spread on top of the macrophage population. Cells were then incubated (37°C and 5% $CO_2$) for 18 hours so that most beads were phagocytosed. The medium was then replaced with high glucose DMEM supplemented with 10% FBS, 10% L929-conditioned media and 1% P/S supplemented with 100 ng/mL crude LPS (InVivogen, San Diego, CA, USA) and 20 ng/mL IFN$\gamma$ (R&D Systems, Abingdon, UK) to polarise macrophages into an M1 state. Medium without LPS and IFN$\gamma$ was used as a control. Cells were incubated (37°C and 5% $CO_2$) for 0, 24 and 48 hours and then fixed with 4°C methanol for 3 minutes and stained with safranin (Sigma-Aldrich, Gilligham, UK) for 10 minutes. The data presented is averaged from four experiments carried with different mice on different days.

### 1.2.3 Lipid accumulation

The protocol was the same for the bead accumulation except without the addition with beads. Cells were fixed prior to (0 hours) and 12, 24, 36, 48 and 60 hours after incubation. Cells were fixed with 4% formalin for 20 mins and stained with oil red O (Sigma-Aldrich, Gilligham, UK) for 15 mins and then stained with Mayer's hematoxylin (Sigma-Aldrich, Gilligham, UK) for 10 mins.

### 1.2.4 Cell quantification

The IncucyteZoom (Sartorius, Göttingen, Germany) was used to obtain the time-lapse microscopy images of bead-loaded macrophage population in 24-well plates. The Hamamatsu Nanozoomer (Hamamatsu Photonics, Japan) was used to obtain whole slide photomicrographs of fixed and stained macrophages. A new in-house image recognition algorithm was developed to quantify either the total number of beads or lipid droplets per cell for every cell in whole slide photomicrographs. The algorithm uses superpixellation and machine learning to learn from a human operator how to differentiate between areas in the image is a bead, lipid droplet, cell or background. Once trained, the algorithm then produces a classification mask across sections of whole slide photomicrographs which identifies whole areas of beads, lipid droplets,

cells or background. A threshold was set for the cell size to filter out other extracellular debris. From this classification mask, the algorithm counts the number of beads per cell (using the average area per bead) or the area of lipid droplets per cell for every cell in the image. This method was used because M1 macrophages strongly adhere to surfaces which precludes cell resuspention and flow cytometry.

## 2 Data accessibility

Data available from the Dryad Digital Repository: https://doi.org/10.5061/dryad.c3269fc. This data includes the whole-slide pictomicrographs of bead and lipid accumulation inside murine macrophage population stimulated with LPS and IFN$\gamma$.

## 3 Acknowledgments

We thank Dan Lucy and Agata Rumianek of the University of Oxford Sir William Dunn School of Pathology for helpful discussions. Mary R. Myerscough and Hugh Z. Ford acknowledge support from an Australian Research Council Discovery Project Grant (to Mary R. Myerscough). David R. Greaves and Hugh Z. Ford acknowledge support from the British Heart Foundation (Programme Grant RG/15/10/31485 to David R. Greaves). We acknowledge the contribution to this study made by the Oxford Centre for Histopathology Research and the Oxford Radcliffe Biobank, in particular: Stephanie Jones, Emma Bowes, Becky Davies, Ying Cui and Clare Verrill.

## 4 Comparison between automatic count by our algorithm and manual counting by human operators

To validate our in-house vision algorithm, an image of bead-loaded macrophages was chosen to be counted (the number of beads per cell) manually by two human operators and automatically by the algorithm. For this we selected one section of a whole slide photo (3%) of the bead-loaded macrophage population 2 days after stimulation with LPS and IFN$\gamma$ (Figure 3 in the main text). A small section of the selected image is displayed in Figure 1. Also shown is the classification mask over the selected image generated by the algorithm. This mask depicts the areas which the algorithm identified as a bead or as a cell. Following specification of the average number of pixels per bead, the algorithm then approximates the number of beads per cell from the number of pixels per cell that it identifies as a bead.

Figure 2A shows the number of cells with $n$ beads counted by the algorithm and both human operators. We see that the number of cells counted with $n$ beads are qualitatively similar for each counting approaches. The variation in numbers counted by each operator is qualitatively similar to the variation in numbers counted by the algorithm. Overall, the total number of cells counted by the algorithm is less than the number of cells counted by the human operators. This disparity arises because the algorithm counts multiple overlapping cells as one, as seen in the classification mask shown in Figure 1B.

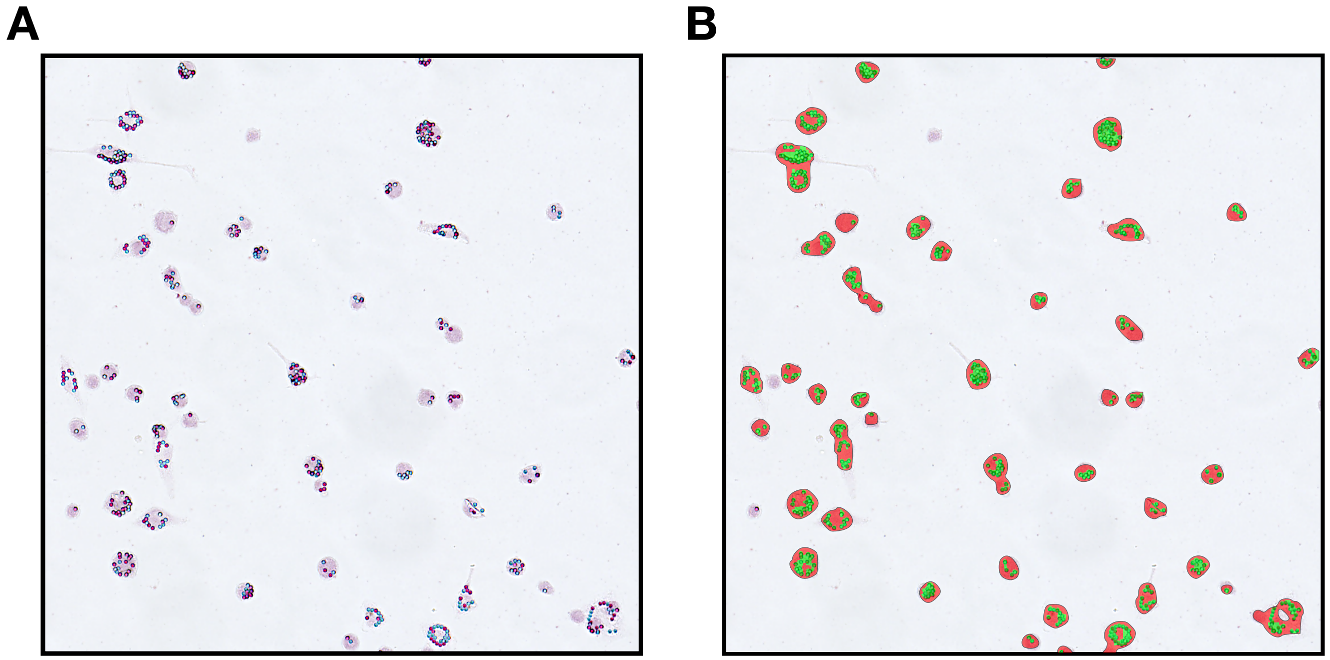


Figure 1: **The algorithm classification mask generated over a representive image.** (**A**) A representative section of the image used to count the number of beads per cell manually by 2 human operators and automatically using the algorithm. (**B**) The classification mask that depicts the areas identified as bead (green) or as cell (red) by the algorithm.

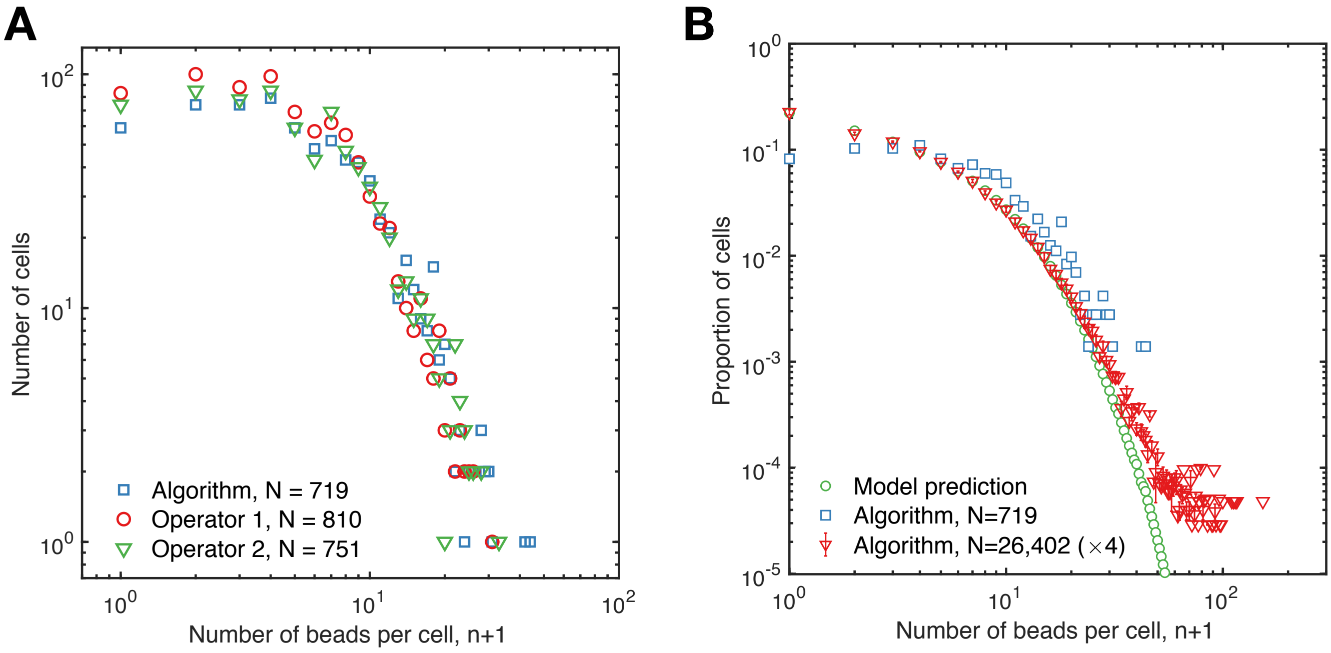


Figure 2: **Comparison between manual and automatic counting (A)** The number of cells with $n$ beads counted by the algorithm (blue squared) and both human operators (red circles and green traingles) in a representative image (a proportion of which is shown in Figure 1). The algorithm counted 719 cells, operator 1 counted 810 cell and operator 2 counted 751 cells. **(B)** The proportion of cells with $n$ beads counted by the algorithm in the representative image (blue squares) or in whole slide photos averaged across 4 experiments (red triangles) compared to the model prediction (green circles). The latter two distributions are derived from Figure 3 in the main text.

Figure 2B shows the proportion of cells with $n$ beads automatically counted from the representative image (Figure 1) compared to the proportion of cells with $n$ beads automatically counted from whole slide photos averaged from 4 experiments (shown in Figure 3 in main text). There are disparities between these distributions. Thee disparities implies that to deduce properties of cell population dynamics, it is necessary to quantify every cell in the population and not sufficient to quantify a select few (even of 100s of cells).

The number of cells with $r$ red beads and $b$ blue beads (total $n = r + b$ beads) counted by the human operators is displayed in Figure 3. The algorithm does not distinguish between red and blue beads. From the proportion of cells with $n$ beads, say $p_n$ (shown in Figure 2), the expected proportion of cells with $r$ red and $b$ blue beads (total $n = r + b$), say $q_{r,b}$, qualitatively follows a binomial distribution:

$$\frac{q_{r,b}}{p_{r+b}} \approx \frac{1}{2^{r+b}} \binom{r+b}{r} = \frac{1}{2^{r+b}} \binom{r+b}{b} . \tag{6}$$

This is a prediction from our mathematical model.

## 5 Estimation of cell death rates

We can estimate the macrophage death rates from the total number of beads counted in each experiment displayed in Figures 3-5 in the main text. We assume that the death rate is constant throughout each experiment. Under this assumption, the mathematical model predicts that the population size $N$ decays exponentially with time $t$ such that:

$$N(t) = N_0 e^{-\beta t} , \tag{7}$$

where $N_0$ cell is the initial number of cells and $\beta$ per hour is the mean cell death rate. Figure 4 shows a fit of this function to the experimental data with estimated parameter values of $N_0$ and $\beta$. The estimated death rates are as follows:

| | LPS+IFN$\gamma$+Beads | LPS+IFN$\gamma$ | LPS+STPN |
|---|---|---|---|
| Death rate, $\beta$ (hours) | 1/60 | 1/80 | 1/40 |

These findings imply that bead loaded macrophages die 1.5 times as fast than those without beads and that LPS and STPN stimulated macrophages die twice as fast as those stimualted with LPS and IFN$\gamma$.

The fact that cell numbers do not exponentially decrease with time implies that the cell death rate does not remain constant throughout the experiment. The death rate is likely to increase with time as nutrients in the media are exhausted and apoptotic factors generated by macrophages, such as tumour necrosis factor, accumulate in the media. Furthermore, as discussed, the the macrophage cell death rate increases as they accumulate lipid and beads [3]. This might have interesting serial killing effects where a cell that consumes a dead cell that has died due to lipid toxicity which also be likely to die via lipid cytotoxicity [4]. Future studies will consider mathematical models which consider the macrophage death rate to be a function of quantity of accumulated substances.

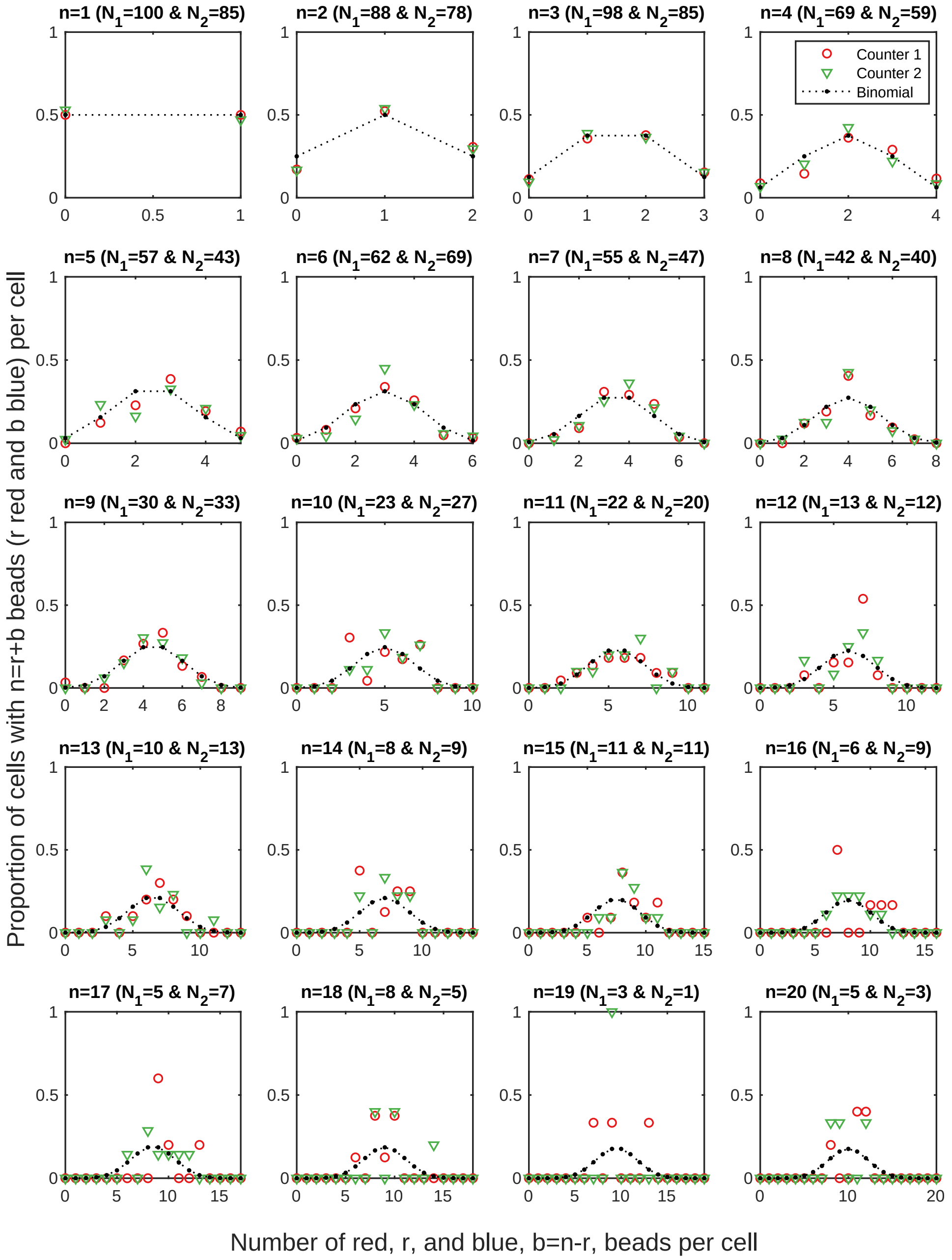


Figure 3: **Red and blue beads are binomially distributed within cells.** The proportion of cells with $n$ beads that contain $r$ red and $b$ blue beads ($n = r + b$) as counted by each human operator (red and green) compared to the binomial distribution (black) given by equation (6). Each plot displays the distribution of cells across $r$ (with $b = n - r$) for different $n$ values ranging from $n = 1$ to $n = 20$ beads (shown in the titles). The title of each plot also displays the number of cells with $n$ beads counted by each operator ($N_1$ and $N_2$).

# 6 Coagulation-fragmentation model derivation

Here, we derive coagulation-fragmentation equations that model the accumulation of beads (and other indigestible substances) inside macrophage populations.

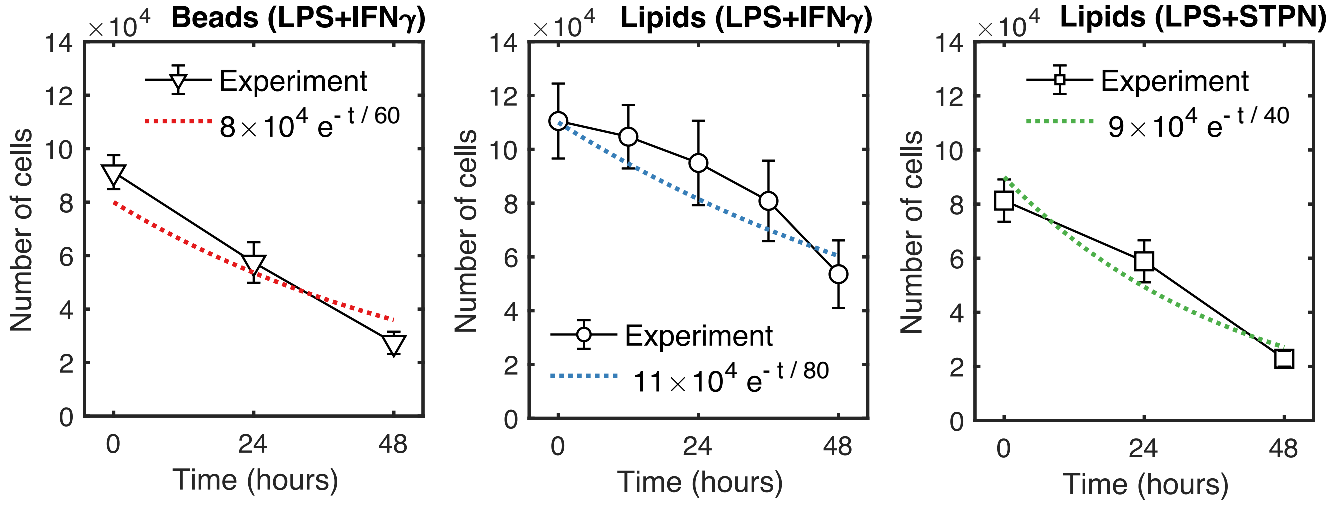


Figure 4: **Estimation of cell death rates.** Fit of an exponential function (equation (7)) to the change in cell numbers seen experimentally for bead loaded macrophages stimulated with LPS and IFN$\gamma$ (left), macrophages stimulated with LPS and IFN$\gamma$ (middle) and macrophages stimulated with LPS and STPN (right). The estimated parameters that represent the initial cell numbers $N_0$ cells and the cell death rate $\beta$ per hour are shown in the figure legends.

## 6.1 Model statement

Let continuous dependent variables $\phi_n(t) \geq 0$ and $\phi_n^\dagger(t) \geq 0$ respectively represent the number density of live and dead macrophages with $n \geq 0$ beads (discrete independent variable) at time $t \geq 0$ (continuous independent variable). Let $\phi(t) \equiv \{\phi_1(t), \phi_2(t), \dots\}$ and $\phi^\dagger(t) \equiv \{\phi_1^\dagger(t), \phi_2^\dagger(t), \dots\}$. We model the time evolution of $\phi_n(t)$ and $\phi_n^\dagger(t)$ via the following system of coupled non-linear ordinary differential equations:

$$\frac{d}{dt}\phi_n(t) = D_n - D'_n - A_n + E_n - E'_n\,, \tag{8}$$

$$\frac{d}{dt}\phi_n^\dagger(t) = A'_n - E''_n\,, \tag{9}$$

where the positive functions are summarised in the following table:

| **Function** | $D_n$ | $D'_n$ | $A_n$ | $A'_n$ | $E_n$ | $E'_n$ | $E''_n$ |
|---|---|---|---|---|---|---|---|
| **Process** | Division | Division | Apoptosis | Apoptosis | Efferocytosis | Efferocytosis | Efferocytosis |
| **Reaction** | Source | Sink | Sink | Source | Source | Sink | Sink |
| **Cell state** | Live | Live | Live | Apoptotic | Live | Live | Apoptotic |

The total numbers of live $N(t)$ and dead $N^\dagger(t)$ cells are given by:

$$N(t) \equiv \sum_{n=0}^{\infty} \phi_n(t) \quad \text{and} \quad N^\dagger(t) \equiv \sum_{n=0}^{\infty} \phi_n^\dagger(t)\,. \tag{10}$$

We denote the initial number of live and dead cells as $N(0) = N_0$ and $N^\dagger(0) = N_0^\dagger$ respectively. We close equations (8) and (9) by assuming that $\Phi_n$ live and $\Phi_n^\dagger$ dead cells initially contain $n$ beads such that:

$$\phi_n(0) = \Phi_n \text{ and } \phi_n^\dagger(0) = \Phi_n^\dagger\,. \tag{11}$$

### 6.2 Specifying the functional forms

The functional forms for $A$, $A'$, $E$, $E'$, $E''$, $D$ and $D'$ that appear in equations (8) and (9) are now introduced.

#### 6.2.1 Apoptosis

We assume that apoptosis: (i) occurs at a constant rate $\beta$ per unit time, (ii) is independent of bead content and (iii) conserves bead numbers. So in equations (8) and (9) apoptosis is modelled by the following equal and opposite sink and source terms:

$$A_n = A'_n = \beta\phi_n(t)\,. \tag{12}$$

#### 6.2.2 Efferocytosis

We assume that efferocytosis (i) occurs at a constant rate $\eta$ per cell per unit time, (ii) is independent of bead content and (iii) conserves bead numbers. So in equation (9) efferocytosis is modelled by a sink term for the number of dead cells which is equal to the rate at which dead cells containing $n$ beads are consumed by any live cell:

$$E'' = \eta\phi_n^\dagger(t)\sum_{n'=0}^{\infty}\phi_{n'}(t) = \eta\phi_n^\dagger(t)N(t)\,. \tag{13}$$

In equation (8) we account for efferocytosis via two terms. A sink term $E'$ that represents the rate at which live cells containing $n$ beads consume dead cells containing $n' = 1, 2, \ldots$ beads:

$$E' = \eta\phi_n(t)N^\dagger(t)\,. \tag{14}$$

The source term $E$ is modelled by a discrete convolution which enumerates all the ways in which a live cell containing $n$ beads can be produced when a live cell containing $n' = 0, 1, \ldots, n$ beads consumes a dead cell containing $'n - n'$ beads:

$$E = \eta\sum_{n'=0}^{n}\phi_{n'}(t)\phi_{n-n'}^\dagger(t)\,. \tag{15}$$

We note that functional form used to model efferocytosis is similar to other experimentally verified models except that we track the quantity of accumulated substances as opposed to the number of consumed apoptotic cells [5, 6]. These models assume, as we do here, that apoptotic cells are consumed whole.

#### 6.2.3 Division

We assume that division: (i) occurs at a constant rate $\alpha$ per unit time, (ii) is independent of bead content, (iii) splits the number of beads contained by the parent cell between two daughter cells and (iv) We each bead inside the parent cell is equally likely to end up in either daughter cell. In equation (8) we account for cell division via two terms A sink term $D'$ that represents the rate at which cells containing $n$ beads divide:

$$D' = \alpha\phi_n(t)\,, \tag{16}$$

The source term $D$ is modelled by the binomial distribution that enumerates the the likelihood that a parent cell with $n' = n, n+1, \ldots$ beads produces daughter cells that contain $n$ beads and $n' - n$ beads [7]:

$$D = 2\alpha \sum_{n'=n}^{\infty} \frac{1}{2^{n'}} \binom{n'}{n} \phi_{n'}(t)\,. \tag{17}$$

### 6.3 The full dimensional model

Substituting equations (12)-(17) into equations (8) and (9) produces the following coagulation-fragmentation equations for $\phi_n(t)$ and $\phi_n^\dagger(t)$:

$$\frac{d}{dt}\phi_n(t) = \underbrace{\eta \sum_{n'=0}^{n} \phi_{n'}^\dagger(t)\, \phi_{n-n'}(t)}_{\text{efferocytosis source}} \underbrace{-\eta\phi_n(t) \sum_{n'=0}^{\infty} \phi_{n'}^\dagger(t)}_{\text{efferocytosis sink}} + \underbrace{\alpha \sum_{n'=n}^{\infty} \binom{n'}{n} \frac{\phi_{n'}(t)}{2^{n'-1}}}_{\text{division source}} \underbrace{-\alpha\phi_n(t)}_{\text{division sink}} \underbrace{-\beta\phi_n(t)}_{\text{apoptosis sink}} \tag{18}$$

$$\frac{d}{dt}\phi_n^\dagger(t) = \underbrace{\beta\phi_n(t)}_{\text{apoptosis source}} \underbrace{-\eta\phi_n^\dagger(t) \sum_{n'=0}^{\infty} \phi_{n'}(t)}_{\text{efferocytosis sink}}\,. \tag{19}$$

The initial condition is given by equation (11).

While this coagulation-fragmentation model is novel, it resembles typical coagulation-fragmentation equations when $\phi_n^\dagger = \phi_n$ and Smoluchowski coagulation equations when $\phi_n^\dagger = \phi_n$ and $\alpha = \beta = 0$ [8–11].

### 6.4 Two differently coloured beads

In our experiments we also considered two differently coloured beads (red and blue). It is straightforward to extend equations (18) and (19) to account for differently coloured beads.

Let $\psi_{i,j}(t)$ and $\psi_{i,j}^\dagger(t)$ resepctively represent the number of live and dead cells that contain $i \geq 0$ red beads and $j \geq 0$ blue beads at time $t$. As such, $\phi_n(t)$ represents the number of cells that contain a total of $n = i + j$ beads (these may be red or blue) so that:

$$\phi_n(t) = \sum_{i+j=n} \psi_{i,j}(t) = \sum_{n'=0}^{n} \psi_{n',n-n'}(t)\,. \tag{20}$$

As for the case with one bead type, the kinetics of apoptosis, efferocytosis and division are given by:

$$\psi_{i,j} \xrightarrow[\beta]{\text{apoptosis}} \psi_{i,j}^\dagger$$
$$\psi_{i,j}^\dagger + \psi_{i',j'} \xrightarrow[\eta]{\text{efferocytosis}} \psi_{i+i',j+j'}$$
$$\psi_{i,j} \xrightarrow[\alpha]{\text{division}} \psi_{i',j'} + \psi_{i-i',j-j'}\ ,$$

for some $i \geq i' \geq 0$ and $j \geq j' \geq 0$. We assume that the accumulation of differently coloured beads are independent. Using the same arguments made in previous subsections, we deduce that the time-evolution

of $\psi_{i,j}(t)$ and $\psi^\dagger_{i,j}(t)$ are given by the following pair of coagulation-fragmentation equations:

$$\frac{d}{dt}\psi_{i,j} = \eta \sum_{i'=0}^{i}\sum_{j'=0}^{j} \psi^\dagger_{i',j'}\psi_{i-i',j-j'} - \eta\psi_{i,j}\sum_{i'=0}^{\infty}\sum_{j'=0}^{\infty}\psi^\dagger_{i',j'} + \alpha\sum_{i'=i}^{\infty}\sum_{j'=j}^{\infty}\binom{i'}{i}\binom{j'}{j}\frac{\psi_{i',j'}}{2^{i'+j'-1}} - (\alpha+\beta)\psi_{i,j}\ , \quad (21)$$

$$\frac{d}{dt}\psi^\dagger_{i,j} = \beta\psi_{i,j} - \eta\psi^\dagger_{i,j}\sum_{i'=0}^{\infty}\sum_{j'=0}^{\infty}\psi_{i',j'}\ . \quad (22)$$

We close equations (21) and (22) by impoing the following initial condition:

$$\psi_{i,j}(0) = \Psi_{i,j} \quad \text{and} \quad \psi^\dagger_{i,j}(0) = \Psi^\dagger_{i,j}\ . \quad (23)$$

### 6.5 Nondimensionalisation

Here we nondimensionalise equations (18) and (19) and equations (21) and (22). The number density of live and dead cells with $n$ beads are scaled with the total number of live and dead cells respectively so that:

$$p_n(t) \equiv \phi_n(t)/N(t) \ \text{ and } \ p^\dagger_n(t) \equiv \phi^\dagger_n(t)/N^\dagger(t)\,, \quad (24)$$

where $p_n(t)$ and $p^\dagger_n(t)$ are the population density of live and dead cells respectively:

$$\sum_{n=0}^{\infty} p_n(t) = \sum_{n=0}^{\infty} p^\dagger_n(t) = 1\,. \quad (25)$$

Similarly, we let:

$$q_n(t) \equiv \psi_{i,j}(t)/N(t) \ \text{ and } \ q^\dagger_n(t) \equiv \psi^\dagger_{i,j}(t)/N^\dagger(t)\,. \quad (26)$$

The total number of live and dead cells are nondimesnionalised by dividing both by the initial number of live $N_0$ cells:

$$\widetilde{N}(t) \equiv N(t)/N_0 \text{ and } \ \widetilde{N}^\dagger(t) \equiv N^\dagger(t)/N_0. \quad (27)$$

Time is rescaled with $\beta^{-1}$, the mean macrophage lifetime, so that:

$$\widetilde{t} \equiv \beta t\,. \quad (28)$$

Substituting equations (24)-(28) into equations (18), (19) and (11) produces the following system of ordinary differential equations for the time-evolution of the proportion of live and dead cells with $n$ beads:

$$\frac{d}{d\widetilde{t}}p_n + \frac{p_n}{\widetilde{N}}\frac{d}{dt}\widetilde{N} = b\widetilde{N}^\dagger\Big(\sum_{n'=0}^{n} p^\dagger_{n'}p_{n-n'} - p_n\Big) + 2a\sum_{n'=n}^{\infty}\binom{n'}{n}\frac{p_{n'}}{2^{n'}} - (1+a)p_n \quad (29)$$

$$\frac{d}{d\widetilde{t}}p^\dagger_n + \frac{p^\dagger_n}{\widetilde{N}^\dagger}\frac{d}{dt}\widetilde{N}^\dagger = \frac{\widetilde{N}}{\widetilde{N}^\dagger}\Big(p_n - b\widetilde{N}^\dagger p^\dagger_n\Big)\ , \quad (30)$$

with:

$$p_n(0) = \frac{\Phi_n}{N_0} \text{ and } p^\dagger_n(0) = \frac{\Phi^\dagger_n}{N^\dagger_0}\ , \quad (31)$$

and:

$$a = \frac{\alpha}{\beta} \quad \text{and} \quad b = \frac{\eta N_0}{\beta}\ . \quad (32)$$

Parameter $a$ represents the rate of division ($\alpha$) relative to the rate of death ($\beta$). Parameter $b$ represents the initial rate of efferocytosis ($\eta N_0$) relative to the rate of death. Hereafter, we neglect the tilde notation.

### 6.6 Solution for population size

In order to determine expressions for $N(t)$ and $N^\dagger(t)$, we note from equation (25) that $\sum_{n=0}^{\infty}\sum_{n'=n}^{\infty}\binom{n'}{n}\frac{p_{n'}(t)}{2^{n'}} = 1$ and $\sum_{n=0}^{\infty}\sum_{n'=0}^{n} p_{n'}(t)p_{n-n'}(t) = 1$. Thus summing equations (29) and (30) across all $n$ yields the following ordinary differential equations for $N(t)$ and $N^\dagger(t)$ respectively:

$$\frac{d}{dt}N = (a-1)N \ , \quad N(0) = 1 \quad \text{and} \quad \frac{d}{dt}N^\dagger = N\Big(1 - bN^\dagger\Big) \ , \quad N^\dagger(0) = \frac{N_0^\dagger}{N_0} \ . \tag{33}$$

which have solutions:

$$N = e^{(a-1)t} \quad \text{and} \quad N^\dagger = \frac{1}{b} + \big(\frac{N_0^\dagger}{N_0} - \frac{1}{b}\big)e^{\frac{b}{a-1}(1-e^{(a-1)t})} \ . \tag{34}$$

As $t \to \infty$ the number of live cells either grows $N(t) \to \infty$, decays $N(t) \to 0$ or remains steady $N(t) = N_0$ when $a > 1$, $a < 1$ and $a = 1$ respectively. As time $t \to \infty$ the number of dead cells either tends to the finite number $\frac{1}{b}$, $\frac{1}{b} + \Big(\frac{N_0}{N_0^\dagger} - \frac{1}{b}\Big)e^{\frac{b}{a-1}}$ or $\frac{N_0}{N_0^\dagger}$ when $a > 1$, $a < 1$ and $a = 1$ respectively.

For simplicity, we assume that the number of dead cells is initially at the steady state value such that it remains constant over time:

$$N_0^\dagger = \frac{N_0}{b} = \frac{\beta}{\eta} \implies N^\dagger(t) = N^\dagger(0) = \frac{N_0^\dagger}{N_0} = \frac{1}{b} \text{ for all } t\,, \ a \text{ and } b > 0 \ . \tag{35}$$

### 6.7 The dimensionless model

Substituting equation (34) into equations (29) and (30) produces the following coagulation-fragmentation equations:

$$\frac{d}{dt}p_n = \sum_{n'=0}^{n} p_{n'}^\dagger p_{n-n'} + 2a\sum_{n'=n}^{\infty}\binom{n'}{n}\frac{p_{n'}}{2^{n'}} - (2a+1)p_n \tag{36}$$

$$\frac{d}{dt}p_n^\dagger = be^{(a-1)t}\Big(p_n - p_n^\dagger\Big) \ . \tag{37}$$

Similarly, the case with two differently coloured beads can be modelled by the following 2D coagulation-fragmentation equation:

$$\frac{d}{dt}q_{i,j} = \sum_{i'=0}^{j}\sum_{j'=0}^{j} q_{i',j'}^\dagger q_{i-i',j-j'} + 2a\sum_{i'=i}^{\infty}\sum_{j'=j}^{\infty}\binom{i'}{i}\binom{j'}{j}\frac{q_{i',j'}}{2^{i'+j'}} - (1+2a)q_{i,j} \ , \tag{38}$$

$$\frac{d}{dt}q_{i,j}^\dagger = be^{(a-1)t}\Big(q_{i,j} - q_{i,j}^\dagger\Big) \ . \tag{39}$$

### 6.8 Analytically tractable model

In general, it is not possible to construct explicit analytical solutions to equations (36) and (37) or equations (38) and (39). However the time-dependent solution can be found analytically for the special case when $a = 0$ (no cell division), $b \to \infty$ (instant apoptotic cell consumption) and $\Phi_n = N_0\delta(n-1)$ and $\Psi_{i,j} = \frac{N_0}{2}\Big(\delta(j-1)\delta(i) + \delta(j)\delta(i-1)\Big)$ where $\delta$ is the Dirac delta function such that $\delta(x) = 1$ if $x = 0$ and $\delta(x) = 0$ (every cell initially contains one bead, half of which are red and the other half blue). From equations (34) and (37), the assumption $b \to \infty$ implies that no apoptotic cells exist $N^\dagger = 0$ and

the proportion of live and dead cells with $n$ beads are equal $p_n^\dagger = p_n$. Thus, under these assumptions, equations (36) and (37) simplify to

$$\frac{d}{dt}p_n = \sum_{n'=1}^{n-1} p_{n'}p_{n-n'} - p_n \ , \quad p_n(0) = \delta(n-1) \ , \tag{40}$$

and equations (38) and (39) simplify to:

$$\frac{d}{dt}q_{i,j} = \sum_{i'=0}^{i}\sum_{j'=0}^{j} q_{i',j'}q_{i-i',j-j'} - q_{i,j} \ , \quad q_{i,j}(0) = \frac{1}{2}\delta(i-1)\delta(j) + \frac{1}{2}\delta(i)\delta(j-1) \ , \tag{41}$$

Note that in this model, the total population size (dimensional) is $N(t) = N_0 e^{-t}$ and the total number of beads inside all cells is $n_{\text{tot}} = N_0$. Therefore the average number of beads per cell exponentially grows with time $n_{\text{avg}}(t) = n_{\text{tot}}/N(t) = e^t$.

Equations (40) and (41) are 1D and 2D coagulation equations. Equation (40) is the Smoluchowski's coagulation equation with the convolution term multiplied by $\frac{1}{2}$. The final solution is slightly different to the solution to Smoluchowski's coagulation equation [9, 11]. Equation (41) is novel, to our knowledge a 2D coagulation equation has not been considered.

# 7 Analytical solutions (no cell division)

## 7.1 1D case: total number of (red and blue) beads per cell

Here we determine the time dependent solution to equation (40). We use a generating function approach used to solve Smoluchowski coagulation equations [9–11]. First, we define:

$$f(z,t) \equiv \sum_{n=1}^{\infty} p_n(t)e^{-nz} \ , \tag{42}$$

noting from equation (40) that:

$$f(z,0) = e^{-z} \ . \tag{43}$$

By the Cauchy product of $f(z,t)$, we have that:

$$f^2(z,t) = \sum_{n=2}^{\infty}\sum_{n'=1}^{n} p_{n'}(t)p_{n-n'}(t)e^{-nz} \ . \tag{44}$$

Using equations (42)-(44), equation (40) supplies the following partial differential equation for $f(z,t)$;

$$\frac{\partial}{\partial t}f = \sum_{n=1}^{\infty}\frac{dp_n}{dt}e^{-nz} = \sum_{n=2}^{\infty}\sum_{n'=1}^{n-1} p_{n'}p_{n-n'}e^{-nz} - \sum_{n=1}^{\infty}p_n e^{-nz} = f^2 - f \ . \tag{45}$$

The solution to equation (45) can be found by (i) integrating with respect to $t$, (ii) applying the initial condition shown in equation (43) and (iii) using the Maclaurin series expansion $\sum_{n=0}^{\infty}(ax)^n = (1-ax)^{-1}$:

$$f(z,t) = e^{-z-t}\Big(1 - (1-e^{-t})e^{-z}\Big)^{-1} = e^{-t}\sum_{n=1}^{\infty}\Big(1-e^{-t}\Big)^{n-1}e^{-nz} \ . \tag{46}$$

Comparison of equations (42) and (46) then supplies the solution for $p_n(t)$:

$$p_n(t) = e^{-t}\Big(1 - e^{-t}\Big)^{n-1} . \tag{47}$$

As sketch of $p_n(t)$ is shown in Figure 5. We can rewrite equation (47) in terms of $N$ and $N_0$ using equation (34). Substituting $t = -\log(N/N_0)$ into equation (47) produces the following geometric distribution:

$$p_n\Big(\frac{N}{N_0}\Big) = \frac{N}{N_0}\Big(1 - \frac{N}{N_0}\Big)^{n-1} . \tag{48}$$

This expression specifies the proportion of cells with $n$ beads when the population size has decreased from $N$ to $N_0$. It is useful for comparison with experimental data.

We can determine the maximum value of $p_n(t)$ for a particular value of $n$ and the time at which the maximum occurs. By setting the first derivative of equation (47) to zero supplies:

$$\frac{d}{dt}p_n(t) = -e^{-t}\Big(1 - e^{-t}\Big)^{n-1} + (n-1)e^{-2t}\Big(1 - e^{-t}\Big)^{n-2} = 0 \implies t = \log(n) . \tag{49}$$

Note that this value of $n$ coincides with the average number of beads per cell $n_{\text{avg}} = e^t$. The maximum value of $p_n(t)$ is given by:

$$\max p_n(t) = p_n\big(\log(n)\big) = \frac{(n-1)^{n-1}}{n^n} = \frac{1}{n}\Big(1 - \frac{1}{n}\Big)^{n-1} \to e^{-1}n^{-1} \text{ as } n \to \infty . \tag{50}$$

### 7.2 Generalisation for two types of beads

We solve equation (41) using the same approach as when there is only one type of bead. In more detail, we introduce:

$$g(x, y, t) \equiv \sum_{i=0}^{\infty}\sum_{j=0}^{\infty} q_{i,j}(t)e^{-ix-jy} . \tag{51}$$

noting from equation (41) that:

$$g(x, y, 0) = \frac{1}{2}\big(e^{-x} + e^{-y}\big) . \tag{52}$$

We note, by the 2D Cauchy product of $g(x, y, t)$, that:

$$g^2 = \sum_{r=0}^{\infty}\sum_{i=0}^{\infty}\sum_{j'=0}^{j}\sum_{i'=0}^{i} q_{i',j'}q_{i-i',j-j'}e^{-ix-jy} . \tag{53}$$

Using equations (51)-(53), we can convert equation (41) into the following partial differential equation:

$$\frac{\partial}{\partial t}g = g^2 - g . \tag{54}$$

The solution to equation (54) can be found by (i) integrating with respect to $t$, (ii) applying the initial condition shown in equation (52) and (iii) using the Maclaurin series expansion:

$$g(x, y, t) = \frac{1}{2}e^{-t}(e^{-x} + e^{-y})\Big(1 - \frac{1}{2}(1 - e^{-t})(e^{-x} + e^{-y})\Big)^{-1} \tag{55}$$

$$= e^{-t}\sum_{k=0}^{\infty}\sum_{\ell=0}^{k+1} \frac{1}{2^{k+1}}(1 - e^{-t})^k \binom{k+1}{\ell} e^{-\ell x-(k+1-\ell)y} . \tag{56}$$

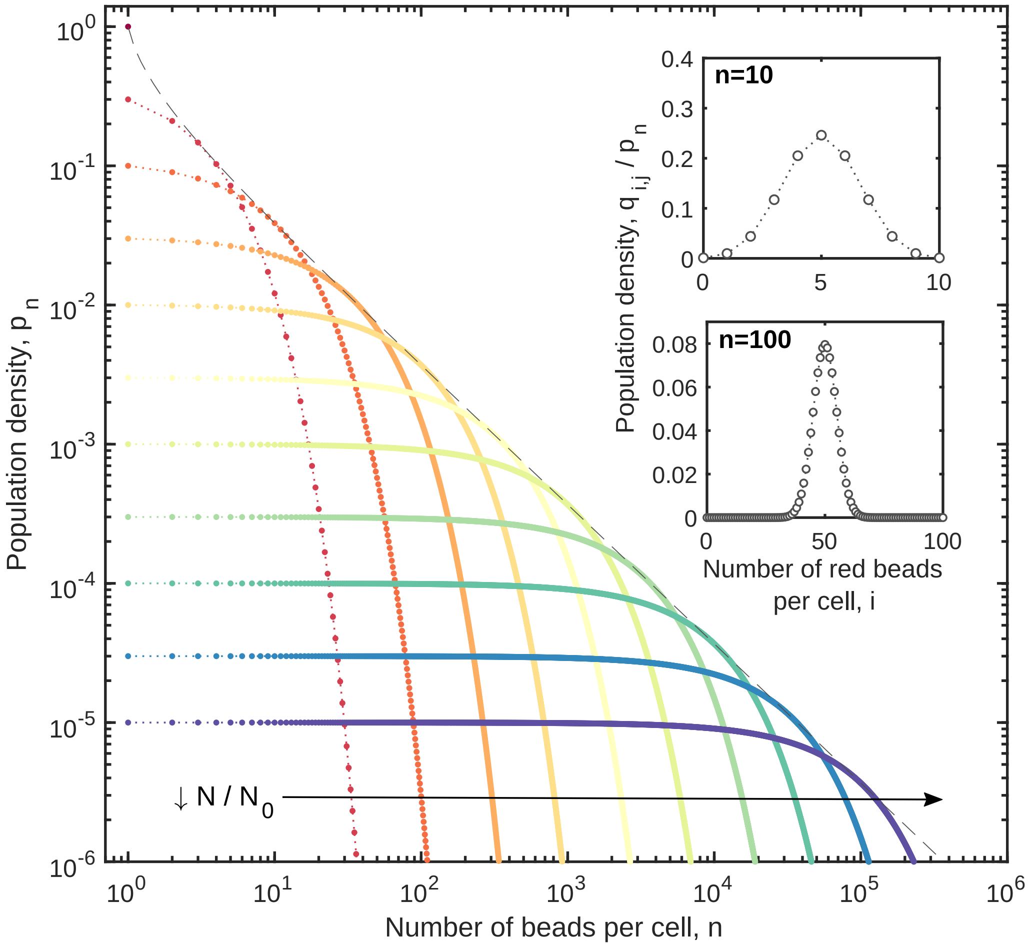


Figure 5: The evolution of the population density distribution across the total number of beads per cell $n$, $p_n$ (equation (48)), and across the number of red beads per cell $i$ and blue beads per cell $j = n - i$, $q_{i,j}$ (equation (58)), as the population size decays $N/N_0 \to 0$. Equation (48) is the analytical solution to equation (40) and equation (58) is the analytical solution to equation (41). Each coloured line represents solution $p_n$ across $n$ when the population size is $N/N_0 = 10^0, 3 \times 10^{-1}, 10^{-1}, 3 \times 10^{-2}, \ldots, 10^{-5}$ from red to blue with the envelope function for $p_n$, $n^{-n}(n-1)^{n-1}$ (dotted grey). The inset plots display the solution for the proportion of the cells with $n$ beads that contain $i$ red and $j = n - i$ blue beads $q_{i,n-i}/p_n = \frac{1}{2^n}\binom{n}{i}$ (binomial distribution) for the case where $n = 10$ (top) and $n = 100$ (bottom).

With $i = \ell$ and $j = k + 1 - \ell$, comparison of equations (51) and (56) reveals that the solution for $q_{i,j}(t)$ is:

$$q_{i,j}(t) = \frac{1}{2^{i+j}}\binom{i+j}{i} e^{-t}(1 - e^{-t})^{i+j-1}, \ q_{0,0}(t) = 0\,. \tag{57}$$

We remark that this expression is equal to the proportion of cells with $n = i + j$ beads $p_{i+j}(t)$ (equation (47)) multiplied by the binomial distribution:

$$q_{i,j} = \frac{1}{2^{i+j}}\binom{i+j}{i} p_{i+j} = \frac{1}{2^{i+j}}\binom{i+j}{j} p_{i+j}\,. \tag{58}$$

The binomial distribution $\frac{1}{2^{i+j}}\binom{i+j}{i} = \frac{1}{2^{i+j}}\binom{i+j}{j}$ gives the likelihood of observing $i$ red and $j$ blue beads inside a cell that contains $n = i + j$ beads. Therefore the solution $q_{i,j}(t)$ is analogous to the solution $p_n(t)$ for $n = i + j$ ($n \geq i, j \geq 0$).

### 7.2.1 Solution description

Figure 5 displays the analytical solution for $p_n$, the proportion of cells with $n \geq 1$ beads, given by equation (48) and $q_{i,j}$, the proportion of cells with $i \geq 0$ red and $j \geq 0$ blue ($n = i+j \geq 1$) beads, given by equation (58). As the population size declines, the population density $p_n$ becomes approximately uniform across a range of $n$ values that grows from $n = 1$.

When a bead content coincides with the average bead content $n = n_{\text{avg}} = e^t$ we have that $p_{n_{\text{avg}}} \approx n_{\text{avg}}^{-1} e^{-1} = e^{-(t+1)}$ (equation (50)) and $p_1 = e^{-t} = n_{\text{avg}}^{-1}$ (equation (47)). Thus the maximum difference in the solution across the domain $1 \leq n \leq n_{\text{avg}}$ exponentially decreases over time $p_1 - p_{n_{\text{avg}}} \approx (e-1)e^{-(t+1)}$. Therefore the population exponentially decays in size approximately as a uniform distribution ($p_n \approx p_1 = e^{-t} = \frac{N}{N_0}$) across an exponentially growing range of number of beads per cell ($1 \leq n < e^t = \frac{N_0}{N}$). That is, the average number of beads per cell and the extent of bead accumulation increases proportional to the decay in population size. And the population heterogeneity (or cell-to-cell variation) with respect to the number of beads per cell also increases as cell numbers decrease.

Furthermore, the population density remains binomially distributed across the number of red $i$ and blue $j = n-i$ beads per cell. Since the number of red $i$ and blue $n = j-i$ beads are distributed binomially, the proportion of red and blue beads per cell become more-or-less equal in cells that accumulate large numbers of beads $n \gg 1$. For example, macrophages with $i = 50$ red and $j = 50$ blue beads are far more likely ($\approx 8\%$) to be observed than macrophages with $i = 75$ red and $j = 25$ blue beads ($\approx 0\%$).

# 8 Case without division and without instant apoptotic cell consumption

We now consider the case where apoptotic cells are not instantly consumed such that model parameter $b$ is finite $b < \infty$. Solutions to equations (36) and (37) were found numerically using the forward Euler scheme [1] as they could not be found analytically. Output from the numerical solutions are displayed in Figure 6 for the cases where $b = 10^1$, $10^3$, $10^2$ and $b \to \infty$.

Figure 6 shows that a reduction in the efferocytosis rate causes a reduction in bead accumulation inside live cells and causes the accumulation of dead cells with low numbers of beads per cell. This is expected as the coalescence of beads via efferocytosis is reduced when the rate of efferocytosis is reduced.

It is possible to find $t$ and $b$ values for when the solutions $p_n(t)$ and $p_n^\dagger(t)$ for $b < \infty$ is well approximated by $p_n(t)$ for $b \to \infty$ (solution given by equation (47)). We find that this approximation holds while $p_n(t) \approx p_n^\dagger(t)$ which, from equation (37), occurs while $be^{-t} \gtrapprox 1 \implies 0 \leq t \lessapprox \log(b)$. This can be seen in Figure 6 which shows that $p_n(t)$ and $p_n^\dagger(t)$ are approximately given by equation (47) at: $t = 1$ for $b = 10^1$ ($\log(b) \approx 2.3$), $t = 1$ and $t = 3$ for $b = 10^2$ ($\log(b) \approx 4.6$) and $t = 1$, 3 and 5 for $b = 10^3$ ($\log(b) \approx 6.9$).

In our experiments we have that $t < 3$ (nondimensionalised time) as the macrophage death rate was estimated as $0.01 < \beta < 0.025$ per hour and our experiments were run over the course of 2 days. Furthermore, we estimate $b = \eta N_0/\beta \approx 10^2$ as we have $N_0 = 10^5$ cells initially in our experiments and the efferocytosis rate is estimated to be $\eta = 10^{-5}$ per cell per hour [5, 6]. Thus we find that the assumption that $\eta \to \infty$ is a reasonable approximation for our experiments, but not for long time periods.

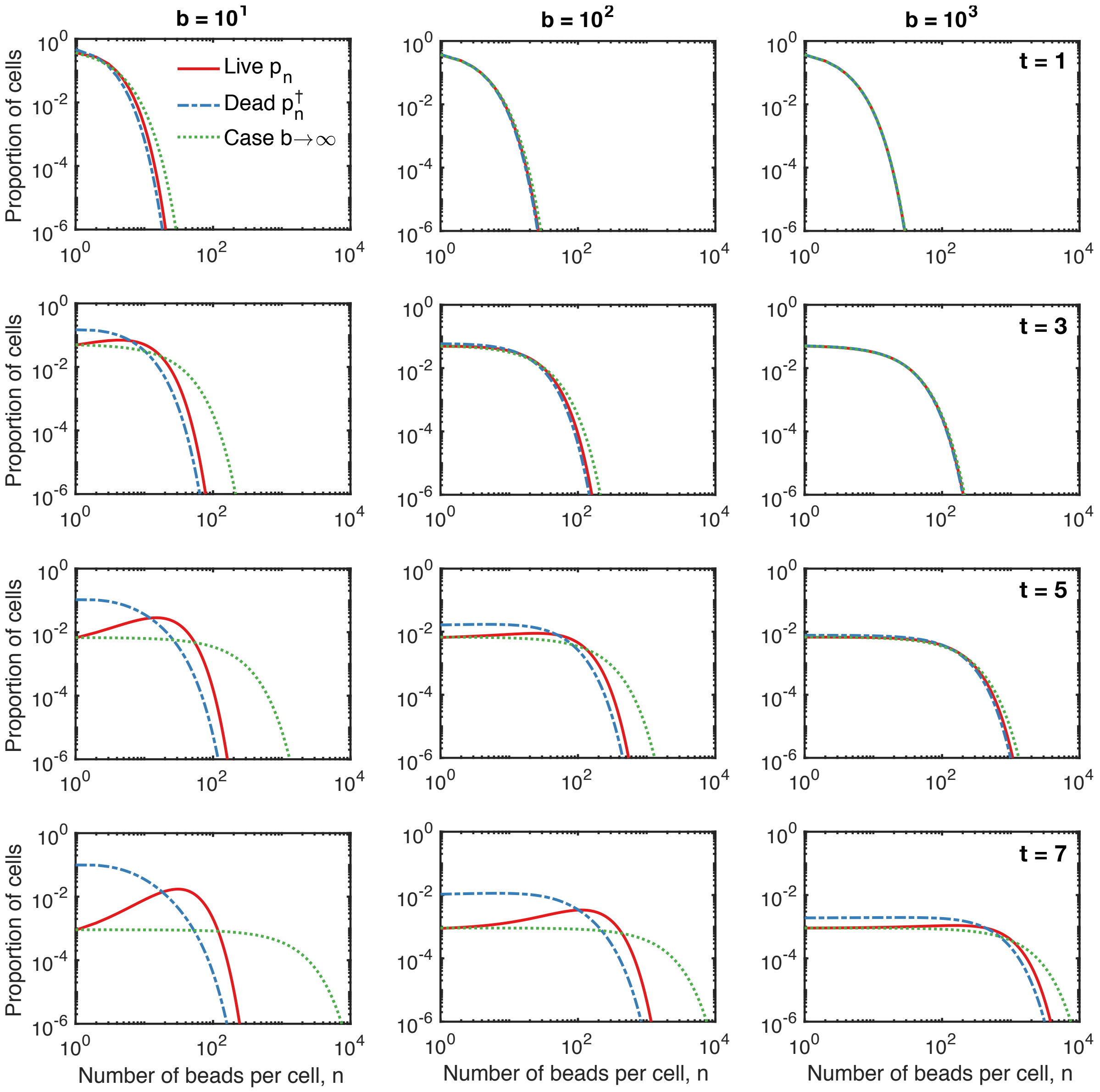


Figure 6: Numerical simulations for time evolution of the proportion of live cells with $n$ beads $p_n(t)$ given by equation (36 (full red) and dead cells with $n$ beads $p_n^\dagger(t)$ given by equation (37) (dashed blue) for $b = 10^3$, $10^2$ and $10^3$ (from left-to-right column) at times $t = 1$, 3, 5 and 7 (from top-to-bottom row). Also shown is the analytical solution to equations (36) and (37) for the time evolution of the proportion of live and dead cells with $n$ beads $p_n(t) = p_n^\dagger(t)$ (dotted green) for the case where efferocytosis is perfect ($b \to \infty$), given by equation (47).

# 9 Case with cell division

We now consider the case with cell division and perfect efferocytosis ($a > 0$ and $b \to \infty$). Numerical solutions to equations (36) and (37) were generated using the forward Euler scheme [1]. Typical results are presented in Figure 7 for cases with: (i) no cell division ($a = 0$), (ii) a cell division rate half of the apoptosis rate ($a = 0.5$), (iii) a cell division rate equal to the apoptosis rate ($a = 1$) and (iv) a cell division rate double the apoptosis rate ($a = 2$).

Figure 7 shows that the dilution of beads via cell division opposes the concentration of beads via apoptosis/efferocytosis. In this way, cell division can stunt, halt or reverse bead accumulation via efferocytosis.

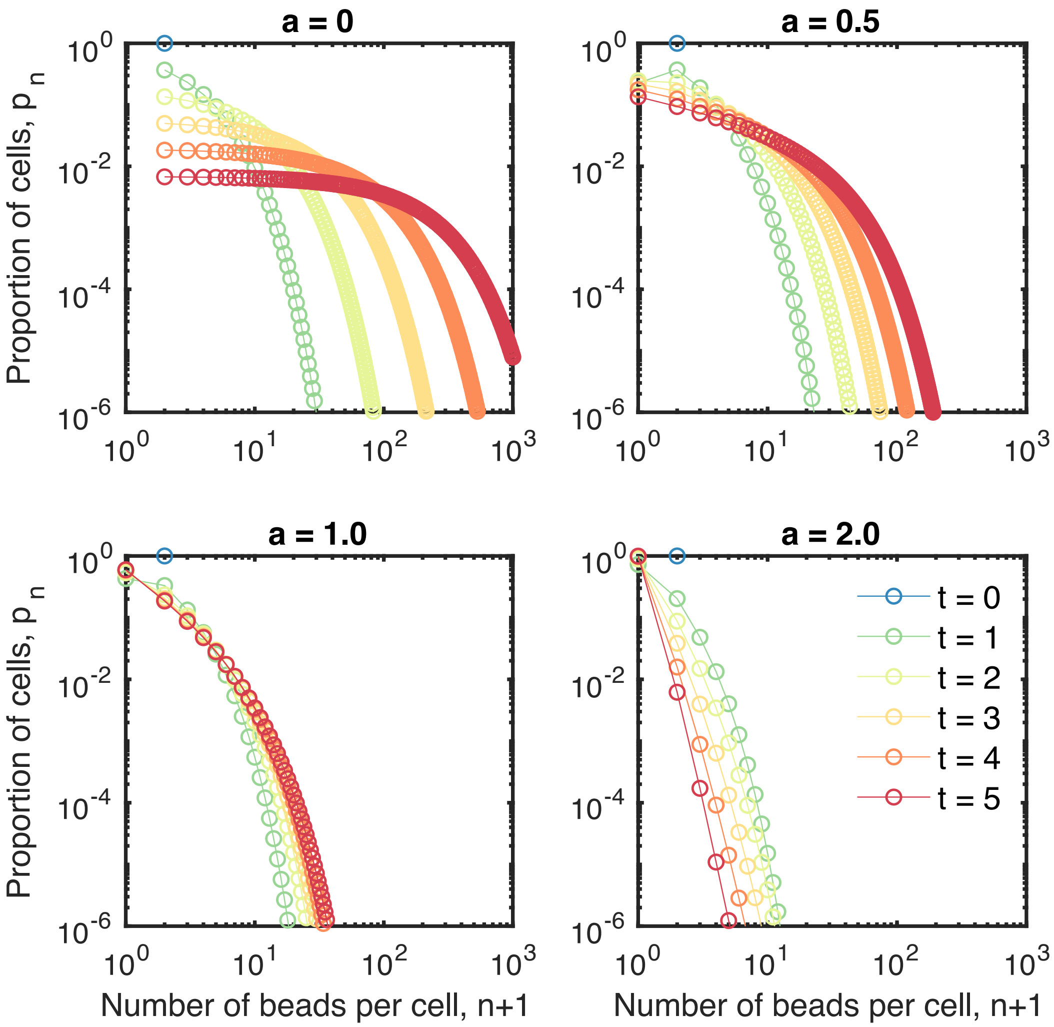


Figure 7: Numerical simulations for time evolution of the proportion of live cells with $n$ beads $p_n(t)$ given by equation (36) for division rate $a = 0$ (top left), $a = 0.5$ (top right), $a = 1$ (bottom left) and $a = 2$ (bottom right) at times $t = 0, 1, \ldots, 5$ (from blue to red).

In our model the number of beads per cell is conserved during cell division, death and efferocytosis such population growth or decay respectively dilutes and concentrates beads inside the population. However, any amount of division ($a > 0$) substantially lowers the extent of bead accumulation in the population compared to the case without cell division ($a = 0$).

When cell division and apoptosis rates are balanced ($a = 1$) macrophage numbers remain constant [12, 13] and the population density tends to a steady distribution. In this state, the beads dynamically redistribute within the cell population while the expected number of cells with $n$ beads $p_n(t)$ remains constant over time. Thus equal division and apoptosis rates produce stability in both the number of macrophages (via balanced cell source and sink effects) and the numbers of beads per cell (via balanced dilution and concentration effects).

The steady state population density distribution is given by the following system of algebraic equations

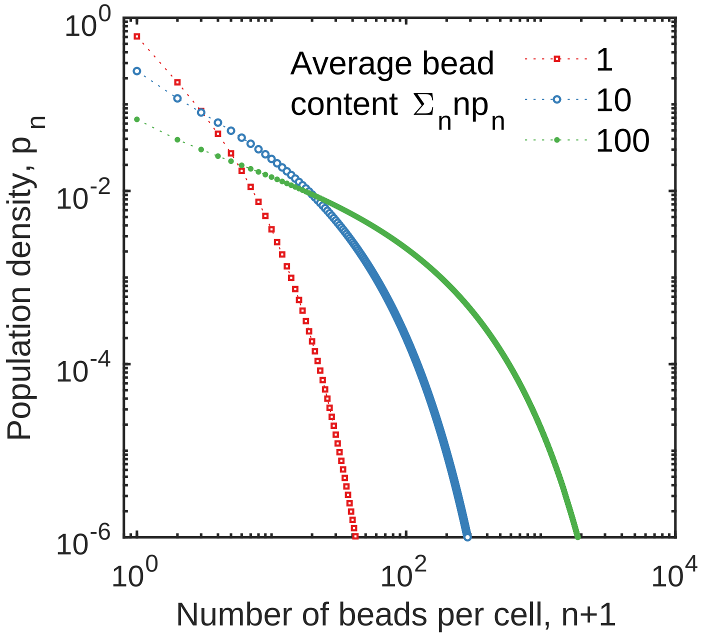


Figure 8: Numerical solutions to equation (59) showing the long time proportion of cells with $n$ beads $p_N$ when there are on average $\sum_{n\geq 0} np_N = 1$ (red squares), 10 (blue open circles) and 100 (green filled circles) beads per cell.

found by setting $a = 1$, $\frac{d}{dt}p_n = 0$ and $\frac{d}{dt}p_n^\dagger = 0$ in equations (36) and (37):

$$3p_n = \sum_{n'=0}^{n} p_{n'}p_{n-n'} + 2\sum_{n'=n}^{\infty} \binom{n'}{n} \frac{p_{n'}}{2^{n'}} . \tag{59}$$

There are an infinite number of solutions to equation (59). The particular solution is set by the total number of beads per cell, given by $\sum_{n\geq 0} np_n$. Solutions to equation (59) can be found using MATLABs nonlinear equation solver. Figure 8 displays these solutions when the average number of beads per cell is $\sum_{n\geq 0} np_n = 1$, 10 and 100. From Figure 8 we see that an increase in the average number of beads per cell increases the extent of bead accumulation inside macrophages. This implies that when the total number of beads inside the population remains fixed,the population can grow in size to obtain a new steady state distribution with a smaller extent of bead accumulation.

In this light, cell division can be viewed as a beneficial mechanism that spreads, maintains and dilutes harmful particles inside macrophage populations.

# References

1. Hamming, R. *Numerical methods for scientists and engineers* (Courier Corporation, 2012).
2. Englen, M., Valdez, Y., Lehnert, N. & Lehnert, B. Granulocyte/macrophage colony-stimulating factor is expressed and secreted in cultures of murine L929 cells. *Journal of immunological methods* **2,** 281–283 (1995).
3. Feng, B. *et al.* The endoplasmic reticulum is the site of cholesterol-induced cytotoxicity in macrophages. *Nature cell biology* **5,** 781–792 (2003).

4. Mahamed, D. *et al.* Intracellular growth of Mycobacterium tuberculosis after macrophage cell death leads to serial killing of host cells. *Elife* **6,** e22028 (2017).

5. Marée, A. F. *et al.* Quantifying macrophage defects in type 1 diabetes. *Journal of theoretical biology* **233,** 533–551 (2005).

6. Marée, A. F., Komba, M., Finegood, D. T. & Edelstein-Keshet, L. A quantitative comparison of rates of phagocytosis and digestion of apoptotic cells by macrophages from normal (BALB/c) and diabetes-prone (NOD) mice. *Journal of applied physiology* **104,** 157–169 (2008).

7. Huh, D. & Paulsson, J. Non-genetic heterogeneity from stochastic partitioning at cell division. *Nature genetics* **43,** 95 (2011).

8. Von Smoluchowski, M. Drei vortrage uber diffusion. Brownsche bewegung und koagulation von kolloidteilchen. *Z. Phys.* **17,** 557–585 (1916).

9. Wattis, J. A. An introduction to mathematical models of coagulation–fragmentation processes: a discrete deterministic mean-field approach. *Physica D: Nonlinear Phenomena* **222,** 1–20 (2006).

10. Bertoin, J. *Random fragmentation and coagulation processes* (Cambridge University Press, 2006).

11. Leyvraz, F. Scaling theory and exactly solved models in the kinetics of irreversible aggregation. *Physics Reports* **383,** 95–212 (2003).

12. Adler, M. *et al.* Endocytosis as a stabilizing mechanism for tissue homeostasis. *Proceedings of the National Academy of Sciences* **115,** E1926–E1935 (2018).

13. Zhou, X. *et al.* Circuit design features of a stable two-cell system. *Cell* **172,** 744–757 (2018).
